# COVID-19-Related Lifestyle Changes among Community-Dwelling Older Adult Day-Care Users: A Qualitative Study

**DOI:** 10.3390/ijerph19010256

**Published:** 2021-12-27

**Authors:** Akira Teramura, Yumi Kimura, Kosuke Hamada, Yasuko Ishimoto, Masato Kawamori

**Affiliations:** 1 Graduate School of Human Sciences, Osaka University, Osaka 565-0871, Japan; yumi621@gmail.com (Y.K.); kosuke2379@gmail.com (K.H.); kawaphon@hus.osaka-u.ac.jp (M.K.); 2 Department of Occupational Therapy, Osaka Health Science University, Osaka 530-0043, Japan; 3 AICHI Medical College for Physical and Occupational Therapy, Kiyosu 452-0931, Japan; 4 Department of Health and Sports Science, Kawasaki University of Medical Welfare, Okayama 701-0193, Japan; i-yasu@mw.kawasaki-m.ac.jp

**Keywords:** COVID-19, older adults, lifestyle change

## Abstract

In Japan, the community-based comprehensive care system is an important initiative. The purpose of this study was to understand COVID-19-related lifestyle changes experienced by older adults who lived in communities and used day-care services. Using a qualitative inductive research method, semi-structured interviews were conducted with 13 older adults who used day-care services in Kyoto City, which assessed lifestyle changes before and after the spread of COVID-19 during March–April 2021. The extracted lifestyle change codes were classified into six categories and 16 subcategories. The data revealed that older adults felt socially isolated and experienced multiple changes in their lives, including limited leisure activities, changes in roles, decreased interpersonal interaction with family and acquaintances, poor diet and sleep quality, and reduced attention to personal appearance and grooming. The findings suggest that during COVID-19, older adults had difficulty adapting to the various changes in their lives and showed a decline in physical and mental functioning. Thus, it is important for day-care facilities to create sustainable spaces in response to the various care needs of community-dwelling older adults whose lifestyles have changed as a result of the COVID-19 situation.

## 1. Introduction

By 2025, the number of people aged 75 and above in Japan is expected to reach 21.8 million [1]. Recently, attention has been focused on preventing older adults from becoming physically dependent and requiring care. The Japanese government is establishing a community-based comprehensive care system so that older adults can continue to live in familiar communities. This system is expected to be established by 2025 and will provide housing, medical care, long-term care, preventive services, and lifestyle support in an integrated manner, while also emphasizing neighborhood relationships [2]. 

On 11 March 2020, the World Health Organization declared a global pandemic caused by COVID-19 [3]. In Japan, as the number of infected people increased, the government notified the public to maintain physical distance and avoid leaving their residences unnecessarily to prevent infection. When a state of emergency was declared, people were discouraged from engaging in community activities [4,5]. Previous studies have indicated that participation in physical and social activities is important to maintain and improve health among older adults [6,7,8]. Such individuals not only face a higher risk of mortality due to COVID-19 but are also more susceptible to impaired physical and mental functioning due to prolonged avoidance of activities and restricted outings.

Quantitative studies performed worldwide have explored the impacts of COVID-19 on the physical and mental functioning of community-dwelling older adults. A study of older adults in Hong Kong reported that the greater the number of days spent at home, the higher the risk of developing anxiety and depression [9]. In Italy, where a lockdown was imposed, older adults with mild cognitive impairment reported worse physical and cognitive functioning [10]. In Brazil, 79% of community-dwelling older adults experienced limited mobility, and those with frailty experienced a two-fold decrease in quality of life compared to those without frailty [11]. In Spanish nursing homes, significant deteriorations were observed in physical and cognitive functioning and nutritional status among 435 older adults, and the authors suggested that interventions against social isolation were required [12]. Thus, restriction of activities to reduce the risk of infection can lead to diminished physical and mental functioning among older adults.

In Japan, most of the previous studies on the impacts of COVID-19 on community-dwelling older adults have been conducted using quantitative approaches. Yamada and colleagues conducted an online survey and found that physical activities of older adults aged 65–84 years and living in urban areas decreased by 65 min per week [13]. Later, they reported that older adults who lived alone and were socially inactive were less likely to improve their physical activity levels [14,15]. Another study also reported that after declaration of the state of emergency in Japan, older adults experienced reduced physical activity, walking time, and took fewer daily steps [16]. Furthermore, a study indicated that the prevalence of depressive symptoms among community-dwelling older adults in Japan had increased slightly during the COVID-19 pandemic [17]. Older adults with frailty have also experienced reduced meal sizes, physical activity, and muscle strength in their lower extremities [18]. Among community-dwelling older adults, the frequency of social activities decreased by 68.1%, along with a decreased frequency in cooking, shopping, and eating out [19]. In contrast, a study of 3199 older adults aged 75 and above found that information and communication technology use was associated with voluntary exercise [20].

A factor that negatively impacted older adults’ social connections and quality of life was the closure of nursing homes and day-care facilities [21]. For older adults who routinely visited day-care facilities before COVID-19, the closure of these facilities meant fewer opportunities to engage in activities outside their homes. A qualitative study conducted six months after COVID-19 first spread through Japan found “distress due to reduced social life” and “concern about a stagnant society” among local residents [22].

However, a year later, there has been no detailed investigation of how COVID-19 affected the daily lives of those aged 75 years or above who used day-care services in Japan. It is necessary to capture the diverse perspectives of older adults to enable the formulation of specific care prevention measures against COVID-19 or similar crises in the future. Therefore, this study aimed to understand the impact of COVID-19 on the lives of persons aged 75 years or above who used day-care services via an interview-based qualitative study. This study may help inform practices that will prevent the need for long-term care post COVID-19 by providing a detailed understanding of the changes experienced by community-dwelling older adults.

## 2. Research Method

### 2.1. Study Design

This study adopted a qualitative research design with semi-structured interviews to understand details of the lives of older adults during the COVID-19 pandemic. The study adhered to the Standards for Reporting Qualitative Research (SRQR) [23]. Ethical approval for this study was obtained from the Research Ethics Committee of the Graduate School of Human Sciences, Osaka University (approval number: OUKSC 20010).

### 2.2. Study Setting and Participants

The study was conducted at a day-care facility located in Nishikyo-Ku, Kyoto City, Kyoto Prefecture. Nishikyo-Ku has several shrines, temples, and ancient tombs, with a nearby historical location for visitors. New residential areas have been developed around the station, and there are parks and walking trails. The aging rate in Kyoto City, which is the ratio of the population over 65 years to the total population, is 28.2%. This aging rate is approximately the same as the national average [24].

The study participants were 13 older adults over 75 years (range 75 to 85 years). They included 3 male participants and 10 female participants who used the day-care service. Approximately 20 people regularly used the facility per week, and the study participants were selected using the snowball sampling method. The exclusion criteria were older adults who had tested positive for or were suspected of having contracted COVID-19, or whose family members were confirmed or suspected of having contracted COVID-19, as this would mean that they were directly affected by COVID-19.

### 2.3. Study Period

The data collection period was from 20 March to 29 April 2021. The principal investigator of this study had been conducting fieldwork at this day-care facility since 2019.

Japan experienced four waves of COVID-19 (March–June 2020, July–October 2020, November 2020–February 2021, and post March 2021); consequently, the number of infections increased drastically, especially among older adults. In Kyoto Prefecture, the study area, a record total of 174 people were newly infected on 24 April 2021. Thus, the study was conducted during and after the fourth wave of the pandemic.

### 2.4. Measurements and Interviews

First, participants’ basic information was collected, including details of sex, age, work experience, family members with whom they lived, and basic activities of daily living (ADL). To assess basic ADL, the participants were interviewed about their independence in seven items (walking, ascending and descending stairs, feeding, dressing, toileting, bathing, and grooming), specifically regarding how much help they required [25]. These were rated from 3 to 0 (3 = completely independent, 2 = requires some help, 1 = requires much help, and 0 = completely dependent), and a full score indicated independence in basic ADL. 

Some measurements were conducted at the study setting: the Mini-Mental State Examination (MMSE) [26], the Timed Up and Go test (TUG) [27], and the Frailty Screening Index (FSI) [28]. The MMSE is generally used to examine the cognitive level of older adults, which is measured from 0–30 points and has 11 questions. The TUG measures the time in seconds it takes to walk 3 m round trip and is effective enough to measure clinical balance in older adults. The cutoff value for the TUG for the risk of falling among community-dwelling older adults is reported to be 13.5 s [29]. The FSI consists of the following five items: “Have you lost two to three kg or more over the past six months?”; “Do you think you walk slower than before?”; “Do you do physical exercise like walking at least once a week?”; “Can you recall what happened five min ago?”; “Have you felt tired for no reason (for the past two weeks)?” Participants were diagnosed as frail if their score was 3 points or above. 

Subsequently, semi-structured interviews were conducted. The interview guide included questions related to participants’ daily life before and after the spread of COVID-19 in January 2021, as well as changes and difficulties experienced during the period when restrictions were in place. Participants were encouraged to talk freely. The interviews were conducted in a quiet place where the privacy of the study participants was assured; interviews lasted 30–60 min. Written informed consent was obtained from the participants after explaining the purpose of the study. With their consent, the interviews were recorded on an integrated circuit recorder and transcribed verbatim for use as descriptive material.

### 2.5. Analysis

A qualitative descriptive analysis, which is widely used in nursing research, was conducted [30]. The following steps were taken: (1) The recorded data were transcribed verbatim and then analyzed; (2) the data were delimited according to their characteristics, and codes were assigned that reflected the content of the delimited data; (3) no framework for analysis was prepared in advance for this survey, and categories were generated from the data itself; (4) these codes were repeatedly examined to account for similarities and differences in the recorded data and were organized and integrated into categories; and (5) data saturation was considered as having been reached when no new codes were obtained from additional interviews. The MAXQDA Analytics Pro ver.2020 (VERBI GmbH, Berlin, Germany) was used to import, organize, and analyze the data.

The reliability and validity of the study were enhanced by discussing the results with three co-investigators who were experts in public health, physical therapy, and sociology in relation to community-dwelling older adults, as well as the principal investigator who has expertise in occupational therapy.

## 3. Results

### 3.1. Overview of the Participants’ Characteristics

Data regarding the 13 participants’ characteristics are presented in Table 1. Seven out of 13 lived alone, four lived with their spouse, and two with their son’s family. All participants were independent based on their ADL scores. The cognitive functions of the participants were relatively well maintained; only two persons among them had a MMSE score of 24 or less, but they had the capacity to answer the interview questions and the other questionnaire without any problems. The frailty scores indicated that 11 participants were frail.

### 3.2. Qualitative Analysis Results

**Figure 1:**
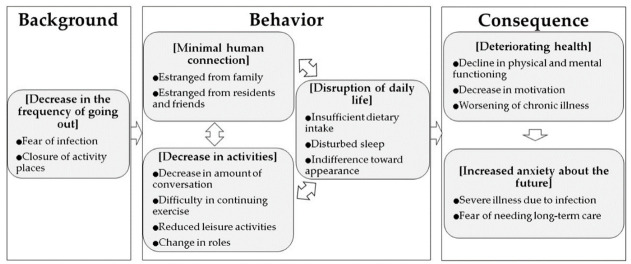
Lifestyle changes experienced by older adults owing to COVID-19.

Through the interview, various life changes were extracted as codes by means of the analysis and classified into six categories; (Decrease in the frequency of going out), (Minimal human connection), (Decrease in activities), (Disruption of daily life), (Deteriorating health), and (Increased anxiety about the future), and 16 subcategories were identified under them. Figure 1 depicts the findings from the analysis. The findings indicate that after the declaration of a state of emergency, participants felt socially isolated due to a decrease in the frequency of going out, and they experienced deteriorating health and increased anxiety about the future due to minimal human connection, decrease in activities, and disruption of daily life. The details of the categories are described below by square brackets, with subcategories identified by double quotation marks and narrative data shared in italics.

#### 3.2.1. Decrease in the Frequency of Going Out

There was a decrease in the frequency of going out due to the impact of COVID-19. This was influenced by psychological factors such as the “fear of infection”, as well as the physical environment, such as “closure of places of activity”.

*In Kyoto, there are many university students because there are many universities and many tourists because it is a tourist area. Young people do not have any symptoms, so I feel that they go around and spread the infection. I do not even like taking the train to go far. When I take the bus, I try not to touch the handrails.*

*(Female)*

*The day-care facility and gyms were closed due to the state of emergency. The facilities have reopened, but I do not feel like going now because I lost the habit of going.*

*(Female)*

#### 3.2.2. Minimal Human Connection

This category included factors such as “estranged from family” and “estranged from residents and friends”.

*I was disappointed that I could not celebrate my grandchild’s birthday or attend a memorial service for my relatives. I feel lonely because I cannot see my family these days.*

*(Female)*

*More than a year has passed without seeing my old friend who lives in another prefecture. We are both very old, so it is a pity that we do not know when we will see each other again, but it cannot be helped. Before the spread of COVID-19, I used to go to restaurants and cafes with my friends from the same day-care service. Now, I do not see them outside the facility.*

*(Female)*

#### 3.2.3. Decrease in Activities

This category included “decreases in amount of conversation”, “difficulty in continuing exercise”, “reduced leisure activities”, and “changes in roles”. That is, for the older adults, the activities they engaged in and the satisfaction they derived from them decreased, and several lifestyle changes were forced on them.

*I did not see my family or acquaintances all day, so I did not have anything to talk about. I can use my cell phone to talk, but it is too complicated to operate.*

*(Female)*

*I tried several ways of exercising at home introduced by some TV programs, but it was difficult for me to continue by myself. I know I need to keep doing the exercises so that I do not become weak, but I cannot make it a habit.*

*(Male)*

*I used to be a member of a haiku poetry club, and I used to take walks with my friends to local places of interest. I have not done it since the COVID-19 spread. I joined a haiku group online, but it is not interesting because the sense of realism is diminished.*

*(Female)*

*I was depressed when I lost the chance to volunteer as a calligraphy instructor, which was one of my favorite activities at the day-care facility.*

*(Female)*

*The salon where my wife with dementia used to go to was temporarily closed, and it was difficult to care for her. I could not take time out for my favorite farm work.*

*(Male)*

#### 3.2.4. Disruption of Daily Life

Disruptions in daily life included “poor eating habits”, “disturbed sleep”, and “indifference toward appearance”. The participants were therefore unable to lead an active daily life as they did before COVID-19.

*Sometimes I ended up eating only boiled rice with tea. I have no appetite, so I eat less often, and some days I eat less than two meals a day. Since I live alone, I am sometimes too lazy to cook.*

*(Female)*

*I live in my loungewear all day long. I do not have a chance to go out, so I do not wear makeup as often. Even when I go out, I wear a mask so I can minimize the amount of makeup I wear.*

*(Female)*

*When I see information related to COVID-19, I sometimes feel anxious about the future and cannot sleep. I do not have to get up in the morning anymore because I do not have the chance to go out for hospital visits or shopping. I do not need to get up in the morning anymore.*

*(Female)*

*There are many days when I sit and watch TV all day and do nothing. All my plans for hospital visits and trips with acquaintances have been canceled, so I am staying at home.*

*(Female)*

#### 3.2.5. Deteriorating Health

This category included negative physical and mental conditions, such as a “decline in physical and mental functioning”, “decrease in motivation”, and “worsening of chronic illnesses”.

*It became difficult to make small movements, such as picking up coins while shopping, and I often forgot what I had to buy. I used to be interested in sewing. I do not feel like sewing, which I used to be interested in.*

*(Female)*

*My family and acquaintances recommended that I make video phone calls with my smartphone, but I do not feel like trying it.*

*(Male)*

*The symptoms of diabetes, which I had before COVID-19, have worsened. My heart palpitations are sometimes intense. In the end, the amount of medication had to be increased to control my chronic disease.*

*(Male)*

#### 3.2.6. Increased Anxiety about the Future

With no end to COVID-19 in sight, there was anxiety about the future, including risk of “severe illness due to infection” and “fear of needing long-term care”.

*There are no hospitals where older adults can be hospitalized.*

*(Female)*

*If I stay indoors and laze around any longer, my body will become too weak, and I will soon need nursing care. It is shameful that I cannot take care of myself anymore.*

*(Male)*

## 4. Discussion

This study aimed to understand the various changes that community-dwelling older adults experienced due to COVID-19. Along with decreases in the frequency of going out, several participants experienced minimal human connection, decrease in activities, and disruption of daily life as behavior changes. Consequently, they dealt with deteriorating health and increased anxiety about the future. A previous survey of community-dwelling older adults in Japan, conducted approximately six months after the spread of COVID-19, reported that they did not experience any changes that would make their lives unsustainable, and they continued with their minimal personal life [22]. However, our study was conducted more than a year after the initial spread of COVID-19, and the participants described significant changes in their lives within their homes and communities, including in relation to interpersonal interactions and leisure activities.

First, the participants experienced minimal human connection. About being “estranged from family”, one participant mentioned that he could not attend his grandchildren’s birthday parties and relatives’ memorial services; not being able to meet family on special occasions heightened his feeling of loneliness. During COVID-19, the family has been an important source of social support for older adults with frailty [31]. In this study, we found that older adults who visited the same day-care service and used to meet outside the facility before the pandemic had fewer opportunities to meet currently, and caused feelings of being “estranged from residents and friends”. As COVID-19 spread further, the loneliness increased, thus careful attention is warranted [32]. Loneliness is associated with an increased risk of early death [33]. Previous studies have shown that older adults who live alone are more likely than those who live with others to feel lonely in the community [34]. A cross-cohort analysis of loneliness before and after the spread of COVID-19 in the United Kingdom also suggested that living alone might increase the risk of loneliness [35]. Consistent with these results, most participants in the present study lived alone and felt isolated because they interacted less with others to prevent becoming infected.

Second, all participants mentioned a decrease in activities in their daily lives. In particular, most of the participants experienced “difficulty in continuing exercise” and “reduced leisure activities”. Owing to COVID-19, the number of people who exercise and the time spent exercising has decreased. TV programs and magazines have introduced home-based exercise regimes. However, our study found that it was difficult for older adults, especially those without exercise habits before the pandemic, to continue exercising through those means. In a qualitative study of the impact of COVID-19 on mental well-being, both young and older adults spent time on hobbies to maintain their motivation for the activity [36]. However, the participants in this study were individuals in the late stages of life (aged 75 years or above) who had difficulty engaging in active leisure pursuits during the pandemic. Lood reported that older adults, aged 85 years or older, who resided in nursing homes in Sweden were similarly constrained in their leisure activities [37]. Thus, given these findings, it is necessary to create places and methods that would allow older adults living at home and in facilities to continue to engage in indoor and outdoor leisure activities.

Within the category of decrease in activities, a noteworthy finding was related to the “change in roles” of the participants. That is, independent and active older adults had to give up their roles of caring for their grandchildren and acting as volunteers in the community. One participant could no longer volunteer as a calligraphy instructor in the day-care facility; thus, she stayed at home, which resulted in minimal activity. Previous studies have shown that the frequency of volunteer participation and interpersonal interactions affects physical and cognitive functioning [38]. Lawton proposed that social roles are of the highest order and involve the most complex abilities, including socializing with people [39]. Older adults with social frailty are at a higher risk of experiencing limitations in ADL and instrumental ADL, which are associated with social roles [40]. However, the restrictions placed on activities to avoid the risk of infection during COVID-19 made it difficult for older adults to maintain such social roles and activities.

Third, the narratives indicated concerns that can be classified under disruption of daily life, with most participants complaining of “insufficient dietary intake” and “disturbed sleep”. The interviews revealed that most participants perceived that they were eating smaller portions than before, tended to consume foods with limited nutritional value, such as fast foods, and boiled rice with tea. During COVID-19, insufficient nutrition intake among older adults has been reported [12], thus more attention should be paid to their diet. Sleep disturbances are likely to occur among older adults, which increase in severity as they grow older. The participants in this study were older than 75 years and experienced difficulties in sleeping, possibly due to decreased daytime activities. Furthermore, since they had fewer opportunities to go out and frequently wore masks, they were less concerned about their appearance and were indifferent toward makeup, clothing, and styling their hair. A significant relationship has been posited between hairdressing and the motivation for rehabilitation among hospitalized patients [41]. We speculate that “indifference toward appearance” might lead older adults to lose the motivation for housework, social activities, and so on.

The interview results revealed that the participants experienced lifestyle changes that were categorized into “minimal human connection”, “decrease in activities”, and “disruption of daily life”, and these factors interacted with each other. Duppen conducted a qualitative study and reported that one of the factors associated of social participation among older adults was changes in the social environment, such as the decline of neighborhood networks [42]. In addition, a previous cohort study in Japan suggested that social frailty might precede physical frailty [43]. Participants in this study were aware that their lifestyle changes had affected their health status. Shinohara reported that, in a questionnaire-based survey of Japanese older adults during the COVID-19 pandemic, frailty status was significantly associated with their self-reported lifestyle changes [44]. For example, one participant in this study explained that she experienced more falls in her house as her knee pain worsened. Another participant faced difficulty with fine motor skills such as taking coins out of her purse. Moreover, most participants said that they felt depressed when they stayed home and had less socialization opportunities with other people because of restricted community activities. Deteriorating health such as “decline in physical and mental functioning”, “decrease in motivation”, and “worsening of chronic illness” lead older adults to feel increased anxiety about the future, where they could become dependent on others and need to be provided sustained care, leading to the “fear of needing long-term care”. The findings of this study indicate the importance of societal role and interaction within the community and how these affect the health status of older adults. It is necessary to consider preventing frailty a social issue to be addressed by the community rather than an issue that individual older adults have to address. Changes and restrictions in the institutional and physical environments can occur not only in the case of a pandemic like COVID-19, but also in emergencies such as other serious pandemics or even disasters. Under these circumstances, this study sheds light on the importance of having support systems that allow older adults to continue social activities at home and within the community, such as hobbies they are interested in and social roles they are good at. Health promotion for older adults, who are a vulnerable population during emergencies, should utilize human networks and social support of family, friends, residents, community volunteers, and medical and welfare professionals to better understand their social activities. In the case of emergencies, more cooperation between human networks in the community will be needed to promote the social participation of older adults. Further research is needed to follow up the situation longitudinally and assess the impact of COVID-19 on older adults’ lives in the context of preventive care.

## 5. Conclusions

The results of this study indicate that the closure of public places and the fear of infection during the COVID-19 pandemic caused not only a decrease in human contact, but also a decrease in activities and a disruption of daily life rhythms among older adults, along with a negative impact on their physical and mental functioning. During emergencies such as pandemics and disasters, older adults would be at a higher risk of frailty as they would be socially isolated and unable to continue their daily activities. Support for older adults to continue social activities that they are good at and social roles they are interested in is important in preventing frailty. In addition, the cooperation of human networks, such as families, residents, local volunteers, and medical and welfare professionals, will be even more necessary during emergencies. This study, which shows the diversity of lifestyles among older adults, might be a useful case study for considering support for vulnerable populations.

## Figures and Tables

**Table 1:** Characteristics of participants.

	Sex	Age (Years)	Work Experience	Family Structure	ADL	MMSE	TUG (s)	FSI
1	Female	80	Housewife	Husband	Independence	30	8.2	4
2	Male	83	Trading company employee	Wife	Independence	24	25.5	3
3	Female	81	Hospital worker	Alone	Independence	30	10.1	2
4	Female	83	Newspaper collector	Alone	Independence	25	27.7	4
5	Female	77	Housewife	Husband	Independence	27	10.8	3
6	Female	83	Housewife	Son’s family	Independence	27	17.1	5
7	Female	78	Housewife	Alone	Independence	30	7.9	3
8	Female	77	Office worker	Son’s family	Independence	28	10.9	3
9	Male	81	Bank clerk	Alone	Independence	25	11.1	3
10	Female	75	Securities clerk	Alone	Independence	25	13.1	4
11	Female	76	Nutritionist	Alone	Independence	30	8.7	3
12	Female	85	Office worker	Alone	Independence	30	8.1	0
13	Male	80	Kimono shop owner	Wife	Independence	22	7.7	2
(Mean ± SD)		79.9 ± 3.1				27.2 ± 2.6	12.8 ± 6.3	3.0 ± 1.1

SD: Standard deviation, ADL: Activities of Daily Living, MMSE: Mini Mental State Examination, TUG: Timed Up and Go Test, FSI: Frailty Screening Index.

## Data Availability

All data generated or analyzed during this study are included in this published article.
